# Extra Virgin Olive Oil Phenols Vasodilate Rat Mesenteric Resistance Artery via Phospholipase C (PLC)-Calcium Microdomains-Potassium Channels (BK_Ca_) Signals

**DOI:** 10.3390/biom11020137

**Published:** 2021-01-21

**Authors:** Rossana D’Agostino, Laura Barberio, Mariacarmela Gatto, Teresa Tropea, Maria De Luca, Maurizio Mandalà

**Affiliations:** 1 Department of Biology, Ecology and Earth Sciences, University of Calabria, 87036 Rende, Italy; roxannedag@gmail.com (R.D.); laura.barberio90@gmail.com (L.B.); mariacarmelagatto91@hotmail.it (M.G.); teresa.tropea@manchester.ac.uk (T.T.); 2 Maternal and Fetal Health Research Centre, Division of Developmental Biology and Medicine, Faculty of Biology, Medicine and Health, University of Manchester, Manchester M13 9WL, UK; 3 Manchester Academic Health Science Centre, Manchester University NHS Foundation Trust, St. Mary’s Hospital, Manchester M13 9WL, UK; 4 Department of Nutrition Sciences, University of Alabama at Birmingham, Birmingham, AL 35294, USA; mdeluca2@uab.edu; 5 Department of Obstetrics, Gynecology and Reproductive Science, University of Vermont, Burlington, VT 05405, USA

**Keywords:** mesenteric artery, BK_Ca_ channels, Ca^2+^ microdomains, PLC, GPCR-Gαi

## Abstract

Recent evidence suggests that the reason Extra Virgin Olive Oil (EVOO) lowers blood pressure and reduces the risk of developing hypertension is partly due to minor components of EVOO, such as phenols. However, little is still known about the mechanism(s) through which EVOO phenols mediate anti-hypertensive effects. The aim of the present study was to investigate the mechanisms of action of EVOO phenols on mesenteric resistance arteries. A pressure myograph was used to test the effect of EVOO phenols on isolated mesenteric arteries in the presence of specific inhibitors of: (1) BKca channels (Paxillin, 10^−5^ M); (2) L-type calcium channels (Verapamil, 10^−5^ M); (3) Ryanodine receptor, RyR (Ryanodine, 10^−5^ M); (4) inositol 1,4,5-triphosphate receptor, IP3R, (2-Aminoethyl diphenylborinate, 2-APB, 3 × 10^−3^ M); (5) phospholipase C, PLC, (U73122, 10^−5^ M), and (6) GPCR-G_αi_ signaling, (Pertussis Toxin, 10^−5^ M). EVOO phenols induced vasodilation of mesenteric arteries in a dose-dependent manner, and this effect was reduced by pre-incubation with Paxillin, Verapamil, Ryanodine, 2-APB, U73122, and Pertussis Toxin. Our data suggest that EVOO phenol-mediated vasodilation requires activation of BKca channels potentially through a local increase of subcellular calcium microdomains, a pivotal mechanism on the base of artery vasodilation. These findings provide novel mechanistic insights for understanding the vasodilatory properties of EVOO phenols on resistance arteries.

## 1. Introduction

Systemic arterial hypertension is the most common preventable risk factor for cardiovascular disease (CVD) [1] and is a leading cause of morbidity, disability, and mortality in the world, with an estimated 17.92 million deaths in 2015 [2]. It is well-established that healthy lifestyle choices, such as diet, exercise, and abstinence from smoking, are associated with a lower risk of CVD [3,4]. In this regard, daily consumption of Extra Virgin Olive Oil (EVOO), a key component of the Mediterranean diet [5], is associated with beneficial effects on human health [6] and, in particular, with reduced cardiovascular risk [7,8]. Epidemiological and clinical studies indicate that daily intake of at least two tablespoons of EVOO can lower blood pressure, thereby decreasing the risk of developing hypertension [9,10,11]. The chemical composition of EVOO includes two different fractions: the saponifiable fraction, which represents 98–99% of EVOO and is characterized by a high content of monounsaturated fatty acid (MUFA), and the unsaponifiable fraction which represents 1–2% of EVOO and includes phenols [12].

The phenolic compounds derive from the secondary metabolism of plants and are chemically characterized by having one or more aromatic rings with one or more hydroxyl groups. The amounts of the phenolic components (150–700 mg/kg) in EVOO depend on many different factors including the cultivars, the degree of maturation, the climate, the production method and the storage [13]. Different groups of phenolic compounds can be found in EVOO, such as secoridoids, phenolic acids, phenolic alcohols, hydroxy-isochromans, flavonoids and lignans [14].

Some studies have associated the anti-hypertensive effect of EVOO to its acidic component, especially oleic acid [15,16]; however, a growing body of evidence suggests that the phenolic component is the major contributor [17,18,19] to the biological effects.

Previously, we showed that EVOO phenols exert a vasodilatory effect on rat resistance mesenteric artery (MA) [20], which may underpin the anti-hypertensive properties of EVOO phenols. We demonstrated that EVOO-phenol-induced vasodilation is endothelium-independent and occurs through activation of the large-conductance calcium-activated potassium (BK_ca_) channels in the vascular smooth muscle cells (VSMCs), but does not involve signaling pathways mediated by nitric oxide (NO)−, prostanoids, or the cyclic nucleotides cAMP and cGMP [20]. The present study will further clarify the mechanism of action of EVOO phenols in MA by investigating the pathways involved in the activation of the BK_ca_ channels.

## 2. Materials and Methods

### 2.1. Extra Virgin Olive Oil Phenols

The phenolic extract used in this work is derived from crude EVOO. The liquid/liquid partitioning method was used with methanol/water (80:20) [21]. The use of methanol/water 80:20 (*v*/*v*) was reported as an efficient extraction solvent [21] and it is used in the official method of phenolic compounds determination. The extraction was performed through the dispersing device UltraTurrax (IKA®-Werke GmbH & Co. KG, Staufen, Germany) for 2 min at 6000 rpm. The obtained emulsion was centrifuged at 4000 rpm for 15 min at room temperature. The hydroalcoholic fraction then was evaporated and later recovered using acetonitrile for residue solubilization and hexane to remove some minor lipid components. The qualitative and quantitative determination of phenolic compounds was accomplished by high performance liquid chromatography (HPLC-UV) [22]. Oleacein (3,4-DHPEA-EDA) is the most abundant phenolic compound in EVOO as well as oleuropein derivatives.

### 2.2. Animals

Male Sprague-Dawley rats (*n* = 16) were used at 18–23 weeks of age. Rats were housed at the University of Calabria under controlled conditions on a 12 h light/dark cycle; commercial chow and tap water were provided ad libitum. All procedures were conducted in accordance with the European Guidelines for the care and use of laboratory animals (Directive 2010/63/EU) and approved by the Italian Institutional Animal Care (n.295/2016-PR). Animals were euthanized with isoflurane and killed by cervical transection; the abdominal cavity was opened immediately, and a section of the mesentery was excised 5 cm distal to the pylorus. The mesenteric tissue was placed in a Sylgard-lined Petri dish containing cold (4 °C) HEPES physiological saline solution (PSS) at pH = 7.4.

### 2.3. Rat Isolated Mesenteric Artery Preparation

Third-order MA were dissected free from the surrounding adipose and connective tissues and cut into segments of about 3 mm in length. Vessels were cannulated in the chamber of a small arteriograph (Instrumentation and Model Facility, University of Vermont, Burlington, VT, USA), superfused with HEPES-PSS at 37 °C and pressurized using a pressure-servo system (Living Systems Instrumentation, St. Albans City, VT, USA). Intraluminal diameter was measured using a video dimension analyzer (Living Systems Instrumentation) and recorded on LabView software.

### 2.4. Experimental Protocol

All arteries were pressurized at an intraluminal pressure of 50 mmHg (as it approximates in vivo conditions) and exposed to K^+^-rich (60 × 10^−3^ M) HEPES-PSS, and Acetylcholine (10 μM), to test artery viability and endothelium integrity, respectively. Vessels that did not elicit reproducible responses to either potassium or acetylcholine were excluded from the study. MA were then equilibrated for 30 min in HEPES-PSS at 37 °C and pre-constricted with phenylephrine to produce a 40–60% reduction in lumen diameter [23]. Increasing concentrations (0.1 ÷ 10 μM) of EVOO phenols were tested on pre-constricted MA, and the resulting changes in diameter were recorded once dilation stabilized at each concentration. At the end of each experiment, vessels were treated with the relaxing solution, HEPES-PSS without Ca^2+^ and containing the phosphodiesterase inhibitor, papaverine (10^−4^ M), to induce maximal vasodilation. To investigate molecular mechanism(s) underlying the EVOO phenol-induced vasodilation, a dose-response curve for phenols (0.1 ÷ 10 μM) was tested in arterial segments pre-incubated for 30 min with one of the following inhibitors, prior to exposure to phenylephrine: (1) Paxillin (10 μM) [20], a selective blocker of BK_ca_; (2) Verapamil (10 μM) [24], a L-type calcium channel blocker; (3) Ryanodyne, (10 μM) [25], an inhibitor of Ryanodine receptor (RyR); (4) 2-APB (3 × 10^3^ μM) [26], an inhibitor of inositol triphosphate receptor (IP3R); (5) U73122 (10 μM) [27], an inhibitor of phospholipase C (PLC); and (6) Pertussis Toxin (10 μM) [28], a blocker of the subunit G_αi_ of G-protein-coupled receptors (GPCR).

### 2.5. Drug and Solutions

The physiological salt solution HEPES-PSS contained the following (in mmol/L): sodium chloride 141.8, potassium chloride 4.7, magnesium sulfate 1.7, calcium chloride 2.8, potassium phosphate 1.2, HEPES 10.0, EDTA 0.5, and dextrose 5.0. The solution was prepared in deionized water and titrated with sodium hydroxide (10 M) to a physiologic pH of 7.4.

K^+^-rich (60 × 10^−3^ M) HEPES-PSS has the same composition as the standard solution, HEPES-PSS, except that NaCl was replaced by an equimolar concentration of KCl.

Chemicals were purchased from Sigma-Aldrich (Merck KGaA, Darmstadt, Germany), Santa-Cruz (Santa Cruz Biotechnology, Inc., Dallas, TX, USA), Cayman Chemical Co (Cayman Chemical, Ann Arbor, MI, USA). unless otherwise specified. All drugs tested were administered from stock solutions prepared daily, except for EVOO phenols stock solutions that were frozen in small aliquots.

### 2.6. Data Analysis and Statistics

Data are expressed as means ± Standard Error Media (SEM), where *n* is both the number of arteries studied and animals used. The vasodilatory effects of EVOO phenols are expressed as percentage of maximal diameter, which was determined in the presence of the relaxing solution. The vasorelaxing efficacy was evaluated as the maximal vasodilatory response (Emax); while the potency was expressed as EC50, calculated as the dilator concentration that caused 50% of the maximum response. Furthermore, we compared the area under the dose-response curve (AUC) at the different experimental conditions. Statistical analysis was performed with paired Student’s *t*-test. *p* < 0.05 was considered significant.

## 3. Results

**Figure 1:**
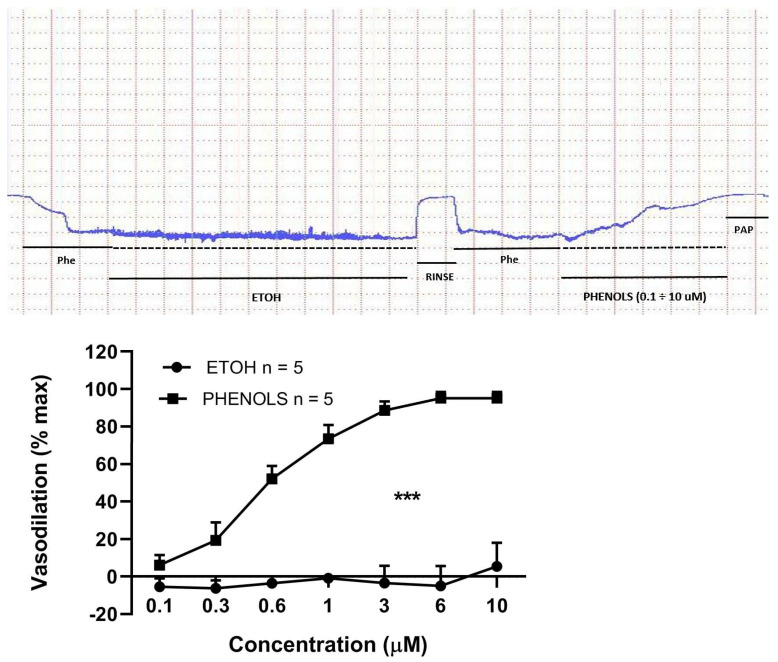
EVOO phenols vasodilation on mesenteric resistance artery. Representative trace showing the effect of Ethanol (EtOH, EVOO phenols vehicle) and EVOO phenols (Phenols, 0.1–10 μM) in phenylephrine pre-constricted mesenteric resistance artery. HEPES-PSS without Ca^2+^, containing Papaverine (Pap) was added at the end of each experiment to calculate maximum dilation. Several experiments were done and summarized in the figure, which shows a dose-dependent vasodilation for EVOO phenols but not for EtOH. Data are presented as the mean ± SEM, n (experimental number), *** *p* < 0.001 refers to the area under the curve.

We tested the effect of EVOO phenols (0.1–10 μM) on MA pre-constricted with phenylephrine. As shown in Figure 1, EVOO phenols induced significant vasodilation (*p* < 0.001) in a dose-dependent manner, with an Emax of 97.74 ± 0.63% and a EC50 of 0.6 ± 0.04 μM. Vehicle condition for EVOO phenols (Ethanol) was assessed and did not show any effect.

**Figure 2:**
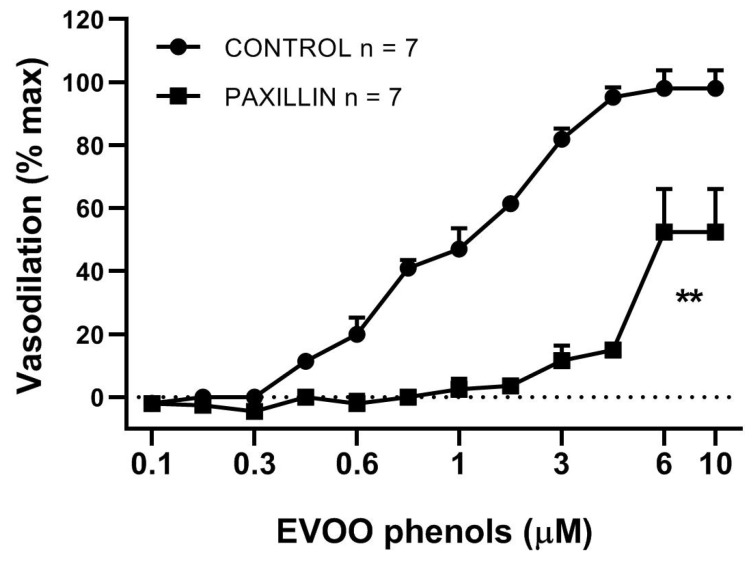
Large-conductance calcium-activated potassium channel blocker reduces EVOO phenol-vasodilation. EVOO phenols were tested on mesenteric resistance artery in the absence (control) and in the presence of the selective inhibitor of the large-conductance calcium-activated potassium (BKca) channels, Paxillin (10 μM). Data are presented as means ± SEM, n (experimental number), ** *p* < 0.01 refers to the area under the curve.

To determine the mechanisms by which EVOO phenols induced vasodilation, a dose-response curve to EVOO phenols was obtained in the presence of the specific inhibitor of BK_ca_ channnels, Paxillin (10 μM) Figure 2. Pre-incubation with Paxillin shifted the dose-response curve to the right, with a significant increasing of EC50 (1 ± 0.08 μM control vs. 5 ± 0.09 μM Paxillin, *p* < 0.01), and reducing of Emax (98.0 ± 5.7% control vs. 52.4 ± 13.6% Paxillin, *p* < 0.05). Interestingly, Paxillin completely abolished EVOO phenol-mediated vasodilation in the dose range of 0.1 μM ÷ 2 μM.

**Figure 3:**
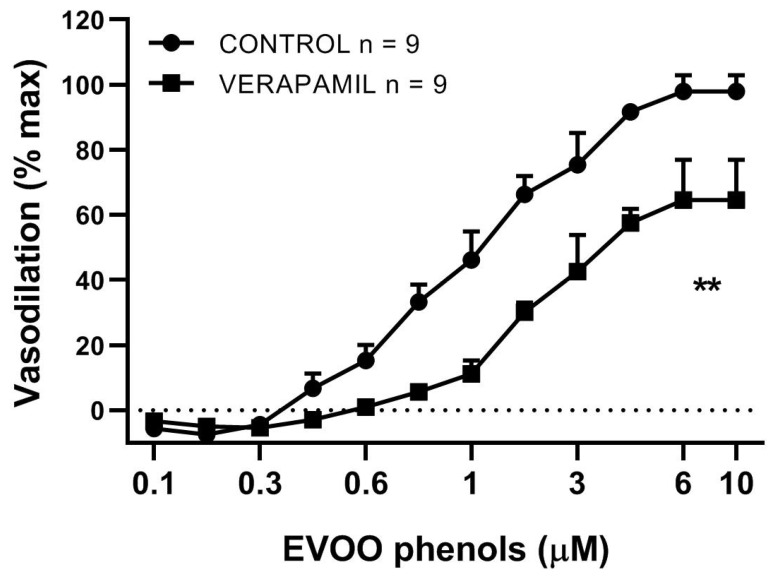
L-type calcium channel blocker attenuates EVOO phenol-vasodilation. EVOO phenols were tested on mesenteric resistance artery in the absence (control) and in the presence of the selective blocker of L-type calcium channel, Verapamil (10 μM). Data are presented as means ± SEM, n (experimental number), ** *p* < 0.01 refers to the area under the curve.

To establish whether EVOO phenol-mediated vasodilation was associated with extracellular Ca^2+^ influx, MA were pre-incubated with the L-type calcium channel blocker, Verapamil (10 μM) Figure 3. By blocking extracellular Ca^2+^ influx, Verapamil induced a rightward shift of the dose-response curve of EVOO phenols, with a significant increase of EC50 (1 ± 0.07 μM control vs. 4 ± 0.24 μM verapamil, *p* < 0.05), and a significant attenuation of Emax (97.9 ± 5.0% control vs. 64.5 ± 12.3% verapamil, *p* < 0.05).

**Figure 4:**
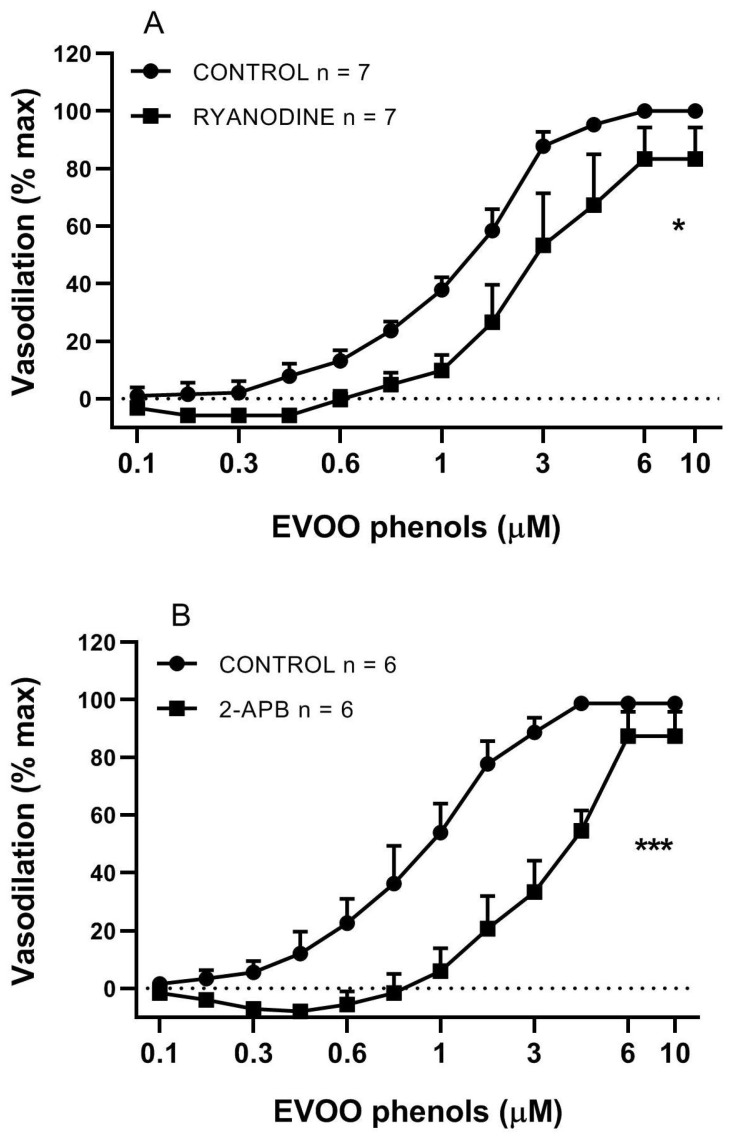
Blockers of intracellular calcium release reduce EVOO phenol-vasodilation. EVOO phenols were tested on mesenteric resistance artery in the absence (control) and in the presence of specific inhibitors of Ryanodine (Ryanodine, 10 μM, **A**) and inositol triphosphate receptors (2-APB, 3 × 10^3^ μM, **B**); Data are reported as means ± SEM, n (experimental number), * *p* < 0.05, *** *p* < 0.001 refer to the area under the curve.

To assess whether the release of Ca^2+^ from intracellular stores was also involved in the vasodilation, a set of experiments was performed using two different blockers of intracellular Ca^2+^ release: Ryanodine (10 μM), a potent inhibitor of RyR, and 2-APB (3 × 10^3^ μM) an inhibitor of IP3R. Ryanodine decreased Emax (98.1 ± 1.53% control vs. 83.3 ± 10.9% ryanodyne, *p* > 0.05), and caused a significant increase of EC50 (1 ± 0.05 μM control vs. 3 ± 0.11 μM Ryanodine, *p* < 0.05), Figure 4A. 2-APB did not significantly affect Emax (98.7 ± 1.2% control vs. 87.3 ± 8.3% 2-APB, *p* > 0.05), but significantly potentiated EC50 (1 ± 0.08 μM control vs. 4 ± 0.04 μM 2-APB, *p* < 0.01), Figure 4B.

**Figure 5:**
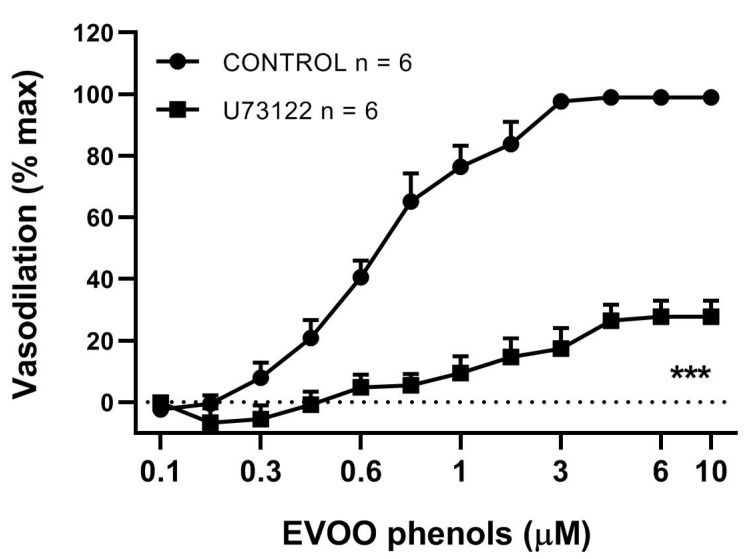
Phospholipase C blocker inhibits EVOO phenol-vasodilation. EVOO phenols were tested on mesenteric artery in the absence (control) and in the presence of the specific inhibitor of phospholipase C, U73122 (10 μM). Data are reported as means ± SEM, n (experimental number), *** *p* < 0.001 refers to the area under the curve.

Furthermore, to confirm the contribution of PLC-initiated in the vasodilatory effect of EVOO phenols, a dose-response curve to EVOO phenols was tested in the presence of the PLC inhibitor U73122 (10 μM), which reduced Emax (98.8 ± 1.1% control vs. 27.7 ± 5,1% U73122, *p* < 0.001) and increased EC50 (0.5 ± 0.06 μM control vs. 1 ± 0.07 μM U73122, *p* < 0.01), Figure 5.

**Figure 6:**
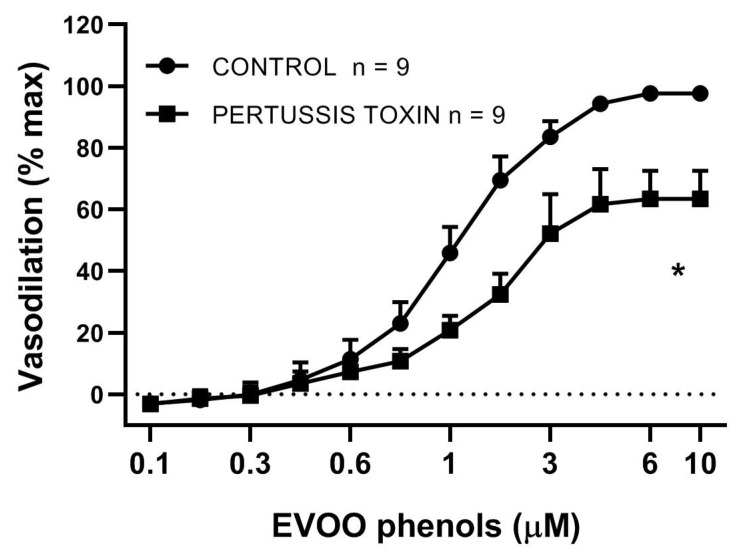
Inactivation of G protein attenuates EVOO phenol-vasodilation. EVOO phenols were tested on mesenteric artery in the absence (control) and in the presence of the specific inhibitor of the GPCR subunit, Gαi, Pertussis Toxin (10 μM,). Data are reported as means ± SEM, n (experimental number), * *p* < 0.05 refers to the area under the curve.

We investigated also whether activation of GPCR is involved in the EVOO phenol-induced vasodilation. Our data showed that inhibition of the subunit G_αi_ of GCPR with Pertussis Toxin (10 μM), significantly decreased Emax (97.6 ± 1.2% control vs. 63.4 ± 9.0% Pertussis Toxin, *p* < 0.05, but did not alter EC50 (1 ± 0.06 μM control vs. 2 ± 0.12 μM Pertussis Toxin, *p* > 0.05), Figure 6.

## 4. Discussion

Our study shows that EVOO phenols dilate resistance MA in a dose-dependent manner. The vasodilation is induced by activation of BK_Ca_ channels through an increase of local intracellular Ca^2+^ level (Ca^2+^ microdomains) as result of influx across the plasma membrane (extracellular Ca^2+^) and release from sarcoplasmic reticulum (SR) Ca^2+^ store. Furthermore, we showed that EVOO phenols activated PLC, which plays a significant role in the regulation of the Ca^2+^ microdomains.

Our results confirm the vasodilation that we showed in our previous study in the same type of artery [20]. A similar effect was reported in rat aortic rings [29,30], suggesting that EVOO phenols are vasodilator of both large (conductive) and small (resistance) vessels. Despite recent studies demonstrated the vasodilatory properties of EVOO phenols, the underlying molecular mechanism(s) of vasodilation are not fully known. A canonical mechanism for the vasodilation is the hyperpolarization mediated by potassium channels. We showed that activation of BK_Ca_ channels is involved in the EVOO phenol-induced vasodilation of MA. In addition, here we demonstrated that both the release of intracellular Ca^2+^ from the SR by IP3R and RyR and extracellular Ca^2+^ influx through the L-type Ca^2+^ channels are implicated in the EVOO phenols vasodilation. Taken together, our results suggest that EVOO phenols activate BK_Ca_ channels potentially through an increase in intracellular localized Ca^2+^ transients. In agreement with other studies, our data suggest that EVOO phenols may lead to the increase of local Ca^2+^ microdomains which, in turn, are responsible for the vasodilatory effect on mesenteric resistance arteries. In muscle cells, the release of Ca^2+^, in the form of Ca^2+^ sparks, induced by RyR in the sub-plasma membrane of the SR, controls the activity of the nearby BK_Ca_ channels in many resistance arteries [31,32,33,34]. On the other hand, Ca^2+^ influx through the L-type Ca^2+^ channels has been shown to activate BK_Ca_ channels in different types of vessels, including hamster and mouse cremaster arterioles [35,36], rat mesenteric arteries [37] and mouse mesenteric arteries [38]. In striated muscle resistance arteries, both Ca^2+^ influx through L-type channels and RyR-based Ca^2+^ sparks, contribute to activation of BK_Ca_ channels [35,36]. Studies in VSMCs from mouse MA [38] have shown that Ca^2+^ influx through L-type Ca^2+^ channels can directly activate BK_Ca_ channels. Furthermore, it has been demonstrated that, in vascular smooth muscle cells, BK_Ca_ and L-type Ca^2+^ channels can directly interact forming complexes that are accumulated in caveolae by the interaction of caveolin-1, thereby regulating spatiotemporal Ca^2+^ dynamics including the negative feedback, to control the arterial excitability and contractility [38].

Furthermore, we demonstrated that EVOO phenols indirectly activated PLC which plays a crucial role in the generation of inositol triphosphate (IP3) a specific agonist of the abundant SR Ca^2+^ channels IP3R. Our results showed that EVOO phenols-PLC activation occurred through the subunit Gαi of GPCR since its inhibition by the specific inhibitor pertussin toxin decreased EVOO phenols vasodilation. This can be explained by the fact that Gαi activation inhibits cAMP-PKA signaling which exerts its inhibitory effects by phosphorylation of both PLC and IP3R, thus limiting the Ca^2+^ mobilization [39].

The involvement of the individual factors PLC, IP3R, RyR and G protein has been reported also in other studies of vasodilation mechanism [40,41,42,43,44,45].

**Figure 7:**
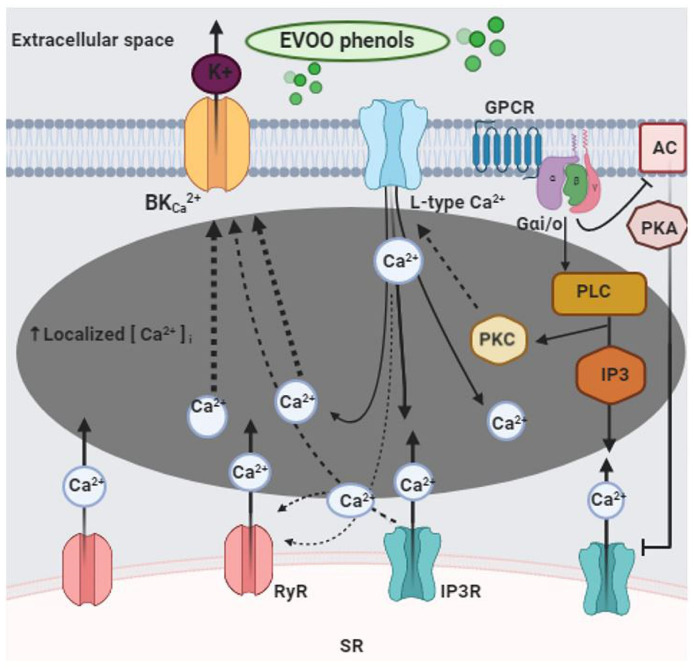
Mechanisms of action of EVOO phenols on vascular smooth muscle cells in mesenteric resistance arteries. EVOO phenols act on the smooth muscle cells of mesenteric resistance artery and trigger intracellular Ca^2+^ signaling. In detail, EVOO phenols activate L-type Ca^2+^ channels and GPCR-Gαi-mediated-PLC pathways, which in turn activate the sarcoplasmic reticulum channels, RyR and IP3R. The overall result of these signaling cascade is the production of localized sites with high concentrations of Ca^2+^, named Ca^2+^ microdomains, which determine the activation of the BKca channels. Calcium Channels L-type (L-type Ca^2+^), large-conductance calcium-activated potassium channels (BKca), inositol 1,4,5-triphosphate receptor (IP3R), phospholipase C (PLC), Ryanodine receptor (RyR), Gαi subunit of G protein-coupled receptor (GPCR-Gαi), protein kinase C (PKC), adenylate cyclase (AC), protein kinase A (PKA), sarcoplasmic reticulum (SR).

In conclusion, the present study contributes to understand the molecular mechanisms of the EVOO phenol-induced vasodilation of MA as summarized in Figure 7. Moreover, it shows, for the first time, that EVOO phenols lower vascular resistance through the activation of the subcellular Ca^2+^ microdomains-BK_Ca_ signal.
